# 重视潜质未定的克隆性造血和意义未定的克隆性血细胞减少症的诊断和治疗

**DOI:** 10.3760/cma.j.cn121090-20241228-00600

**Published:** 2025-01

**Authors:** 志坚 肖

**Affiliations:** 1 中国医学科学院血液病医院（中国医学科学院血液学研究所），血液与健康全国重点实验室，国家血液系统疾病临床医学研究中心，细胞生态海河实验室，天津 300020 State Key Laboratory of Experimental Hematology, National Clinical Research Center for Blood Diseases, Haihe Laboratory of Cell Ecosystem, Institute of Hematology & Blood Diseases Hospital, Chinese Academy of Medical Sciences & Peking Union Medical College, Tianjin 300020, China; 2 天津医学健康研究院，天津 301600 Tianjin Institutes of Health Science, Tianjin 301600, China

**Keywords:** 克隆性造血, 潜质未定的克隆性造血, 意义未定的克隆性血细胞减少症, 诊断, 治疗, Clonal hematopoiesis, Clonal hematopoiesis of indeterminate potential, Clonal cytopenias of undetermined significance, Diagnosis, Treatment

## Abstract

2022年提出的造血淋巴肿瘤WHO分型（WHO 2022）以及髓系肿瘤和急性白血病国际共识分型（ICC）已将潜质未定的克隆性造血（CHIP）和意义未定的克隆性血细胞减少症（CCUS）正式列入了髓系肿瘤分型。最近又相继提出了三个CHIP和CCUS髓系肿瘤转化分险的预测模型，并已开展了CHIP和CCUS干预治疗临床试验。为了规范CHIP和CCUS的诊断和治疗，本文就目前我国面临的挑战以及可能的解决方案做了探讨。

## Abstract (en)

Clonal hematopoiesis of indeterminate potential（CHIP）and clonal cytopenias of undetermined significance（CCUS）are included in both fifth edition of the World Health Organization classification（WHO 2022）and International Consensus Classification（ICC）. Recently three models were developed for prediction of myeloid neoplasms risk and clinical trials are initiated to investigate potential treatments for CHIP and CCUS. Challenges in diagnosis and therapeutic intervention of CHIP and CCUS were discussed.

克隆性造血（Clonal hematopoiesis，CH）是指某个个体不满足任何一种髓系肿瘤诊断标准但有1个与一种髓系肿瘤相关的体细胞突变［变异等位基因频率（VAF）≥2.0%］或细胞遗传学异常[1]–[3]。2022提出的造血淋巴肿瘤（Haematolymphoid tumours）WHO分型（WHO 2022）以及髓系肿瘤和急性白血病国际共识分型（International consensus classification，ICC）均将此前基于CH提出的潜质未定的克隆性造血（Clonal hematopoiesis of indeterminate potential，CHIP）和意义未定的克隆性血细胞减少症（Clonal cytopenias of undetermined significance，CCUS）列入了髓系肿瘤范畴[4]–[5]，为更好地研究这两种新提出的血液系统疾病，国外已成立CCUS联盟（CCUS consortium），有的医院开设了克隆门诊（Clonal clinic），我国应对这两种疾病的诊断和治疗引起高度重视，争取与国际并跑。

一、概念的提出

2014年12月新英格兰医学杂志同一期发表了2篇论著，第一个研究[1]入组人群为12 380名瑞典人，采用外周血标本提取DNA进行全外显子测序，65岁以上人群中10%有伴体细胞突变的CH，50岁以下人群仅为1%，最常检出突变基因有DNMT3A、ASXL1和TET2，CH是一个强的血液系统肿瘤发生危险因素（*HR*＝12.9，95% *CI* 5.8～28.7）。另一研究[2]入组17 182人，采用外周血标本提取DNA进行全外显子测序，70～79岁、80～89岁和90～108岁人群克隆性突变检出率分别为9.5%、11.7%和18.4%，高频突变基因为DNMT3A、TET2和AXSL1，突变显著增加血液系统肿瘤的发生风险（*HR*＝11.1，95% *CI* 3.9～32.6）、全因死亡（*HR*＝1.4，95% *CI* 1.1～1.8）、冠心病（*HR*＝2.0，95% *CI* 1.2～3.4）和缺血性脑卒中患病率（*HR*＝2.6，95% *CI* 1.4～4.8）。这两个大系列人群研究证实年龄相关的克隆性造血（Age-related clonal hematopoiesis，ARCH）是一种常见情况。

2005年在日本长崎召开的第8届国际骨髓增生异常综合征（MDS）研讨会上Mufti教授首次提出了意义未定的特发性血细胞减少［Idiopathic cytopenia of uncertain（undetermined）significance, ICUS］的概念，在2007年发表的“MDS诊断和治疗的定义和标准”共识中正式定义了ICUS[6]，其特征为：①髓系细胞中一系或多系血细胞减少，持续≥6个月：红细胞（HGB<110 g/L）、中性粒细胞（ANC<1.5×10^9^/L）和（或）巨核细胞系（PLT<100×10^9^/L）。②不能满足MDS最低诊断标准。③除外可作为初始原因导致血细胞减少/发育异常的其他所有造血组织或非造血组织的疾病。

基于ARCH的提出，在2015年又相继提出了2个新概念。第1个是CHIP[7]，其主要特征有：①无血液肿瘤细胞形态学证据；②不符合阵发性血红蛋白尿症（PNH）、意义未定的单克隆丙种球蛋白血症（MUGS）和单克隆B淋巴细胞增多症（MBL）的诊断标准；③存在血液肿瘤相关基因突变阳性（VAF≥2.0%）；④每年有0.5%～1%的概率进展为肿瘤。CHIP中那些存在血液肿瘤相关基因突变阳性且VAF<2.0%则称之为ARCH，而那些血液肿瘤相关基因突变为已证实为疾病起始（driver）基因（如JAK2、CALR、MPL、KIT、NPM1和TP53等）的人群又有人将之列为高度致癌潜能的克隆性造血（Clonal hematopoiesis with high oncogenic potential，CHOP）。ARCH和CHIP突变基因常为髓系肿瘤重现性突变基因，2021年提出的淋巴CHIP（lymphoid CHIP，L-CHIP）其突变基因则为淋巴肿瘤重现性基因，与CHIP起源于造血干细胞阶段不同，L-CHIP起源于淋系定向祖细胞。第2个是CCUS[8]，其建议诊断标准为：①髓系细胞中一系或多系血细胞减少，持续≥6个月：红细胞（HGB<110 g/L）、中性粒细胞（ANC<1.5×10^9^/L）和（或）巨核细胞系（PLT<100×10^9^/L）。②骨髓细胞形态学不够MDS发育异常最低诊断标准（红系、粒系和巨核细胞系发育异常细胞均<10%），且无MDS特征性细胞遗传学异常。③存在髓系肿瘤相关基因突变阳性（VAF≥2.0%）或非重现性血液肿瘤染色体异常。④除外可作为初始原因导致血细胞减少/发育异常的其他所有造血组织或非造血组织的疾病。

WHO 2022和ICC已将CHIP和CCUS正式列为髓系前驱性疾病，将CHIP和CCUS诊断标准的血细胞减少定义修订为：贫血（男性HGB<130 g/L，女性HGB<120 g/L），中性粒细胞减少（ANC<1.8×10^9^/L），血小板减少（PLT<150×10^9^/L）。ICC将意义未定的克隆性单核细胞增多症（Clonal monocytosis of undetermined significance，CMUS）也归于CCUS范畴。CMUS的诊断标准为：①持续性单核细胞增多，外周血单核细胞绝对值≥0.5×10^9^/L且白细胞分类计数单核细胞比例≥10%；②有血细胞计数减少（同MDS血细胞减少诊断标准）；③至少有一个髓系肿瘤相关基因突变（VAF≥2.0%）；④无显著的发育异常、原始细胞（包括幼稚单核细胞）增高或骨髓检查有CMML的形态学发现；⑤不符合其他髓系或其他造血肿瘤诊断标准；⑥无其他可以解释反应性单核细胞增多的疾病。

二、CHIP和CCUS髓系肿瘤的转化分险评估系统

CHIP和CCUS的一个主要临床特征是进展为髓系肿瘤风险明显增高，如何来预测CHIP和CCUS患者的髓系肿瘤转化分险是一个最为关注的科学问题。最近已相继提出了三个髓系肿瘤转化分险预测模型。

Weeks等[9]从U.K. Biobank（UKB）数据库2006年至2010年间502 490名40～70岁的参与者中调取了50 834名的资料，最终438 890名纳入研究，将其中193 743名作为建模组，245 147名作为验证组，提出了一个克隆性造血预后积分（Clonal hematopoiesis risk score，CHRS）系统（表1）。根据积分分为三个分险组：高危，≥12.5分；中危，10～12分；低危，≤9.5分。高危、中危和低危髓系肿瘤累计发生率分别为（52.20±4.96）%、（7.83±0.807）%和（0.669±0.0827）%。CHRS的应用场景已在2个真实世界研究得到了确证[10]–[11]。

**表1:** 克隆性造血预后积分（CHRS）[9]

预后参数	分值				
	0.5	1.0	1.5	2.0	2.5
单个DNMT3A突变	有	无			
高危突变^a^		无			有
突变数目		1		≥2	
VAF		<0.2		≥0.2	
红细胞分布宽度		<15			≥15
平均红细胞体积（fl）		<100			≥100
血细胞减少		CHIP	CCUS		
年龄（岁）		<65	≥65		

**注** ^a^ 包括SRSF2、SF3B1、ZRSR2、IDH1、IDH2、FLT3、RUNX1和JAK2基因突变；CHIP：潜质未定的克隆性造血；CCUS：意义未定的克隆性血细胞减少症；VAF：变异等位基因频率

Gu等[12]同样用UKB数据库的454 340名参与者资料进行了另一个研究，中位随访7.9年，1937例在随访期确诊了髓系肿瘤，其中急性髓系白血病（AML）372例，MDS 517例，骨髓增殖性肿瘤（MPN）892例，慢性粒-单核细胞白血病（CMML）27例。确诊了髓系肿瘤的人群中28.5%有CH基因突变，且常为“高危”基因突变，如JAK2、SRSF2、SF3B1和IDH2，而没有发生髓系肿瘤的人群仅4.8%检出CH基因突变，且突变基因常为DNMT3A、TET2和ASXL1。利用基因突变和血液学参数构建了一个COX回归“MN预测（MN-predict）”模型，在网页（https://bioinf.stemcells.cam.ac.uk/shiny/vassiliou/MN_predict）输入相关参数后可预测CH的髓系肿瘤发生风险。MN-predict为一动态风险预测模型，可预测不同时点的髓系肿瘤发生分险。

Xie等[13]收集了真实世界的357例CCUS患者，提出了一个克隆性血细胞减少危度积分（the clonal cytopenia risk score，CCRS）：有剪切基因突变，2分；PLT <100 × 10^9^/L，2.5分；≥2个突变，3分。根据累计积分将患者分为低危（<2.5分）、中危（2.5至<5分）和高危（≥5分）三组，2年髓系肿瘤累计发生率分别为6.4%、14.1%和37.2%。

三、CHIP和CCUS的治疗干预

CHIP和CCUS的治疗干预现今主要依据CHRS预后分组，所有患者都应进行心脑血管梗死危险因素评估，定期监测血常规和白细胞分类计数（高危组每3～6个月1次，中危和低危至少每年1次），高危组患者每年应监测一次二代测序（NGS）和在临床提示病情进展时复查骨髓穿刺，中危和低危组在临床提示病情进展时才复查NGS和骨髓穿刺[14]。

国外已启动治疗干预临床试验[15]–[16]，如抗IL-1β卡那单抗（canakinumab）治疗高危CCUS（NCT05641831）、维生素C治疗TET2-CCUS（NCT03418038）和采用IDH1/2抑制剂来探索IDH1/2-CCUS（NCT05030441和NCT05102370）的靶向治疗。但如何来制定临床试验的入组标准和临床试验的研究终点等尚无国际共识，最近一组美国专家发表了基于专家共识的CH治疗干预和早期临床试验的蓝皮书（blueprint），就临床试验设计要素、患者入组标准和研究终点做出了推荐，值得参考借鉴[17]。

四、我国CHIP和CCUS诊治面临的挑战与解决方案建议

从首次提出CHIP到将CHIP和CCUS正式列入髓系肿瘤的分型诊断已有10年，为了使这些患者获得同质化、规范化诊治，笔者认为在我国以下几点值得高度重视。

其一，基因突变检测是CHIP和CCUS得以确诊的前提，在我国血液科自建实验室和第三方检测实验室开展基因检测的套餐（panel）不尽一致，在WHO（2022）诊断标准中将DNMT3A、TET2、ASXL1、JAK2、TP53、SF3B1、PPM1D、SRSF2、ZBTB33、IDH1、IDH2、U2AF1、KRAS、NRAS、CTCF、CBL、GNB1、BRCC3、PTPN11、GNAS、BCOR和BCORL1作为诊断CHIP和CCUS的必检基因，因此，基因检测套餐中应包括以上基因。

其二，尽管国际上已提出CHRS、MN-predict和CCRS三个髓系肿瘤转化风险预测模型，但这些风险预测模型是基于西方人群提出，CCRS则主要基于大型研究型医院患者数据且随访时间较短（中位随访时间27.3个月），此外，我们比较了本中心与MDS国际预后工作组（IWG-PM）MDS患者临床和实验室特征，发现我国MDS患者较IWG-PM队列患者发病年龄更年轻（中位年龄56岁对72岁，*P*<0.001），三系血细胞计数更低（*P*<0.001），染色体核型出现单独del（20q）、+8和复杂核型的比例较高，正常核型、del（5q）、-Y比例较低（*P*<0.001），U2AF1、NRAS、NPM1基因突变率较高（*P*<0.05），而ASXL1、SF3B1、RUNX1等突变率较低（*P*<0.05）。基于西方人群的髓系肿瘤风险预测模型是否同样适用我国患者有待确证，由于迄今尚无我国CHIP和CCUS患者队列数据，因此，亟待收集我国多中心CHIP和CCUS患者数据来验证这三个危度积分系统是否适用于我国患者，如果不适用则应构建和提出基于我国患者数据的预后风险模型。

其三，CH的治疗干预是一个处于起步阶段的研究领域，国际上已开展了一些临床试验，我国应充分利用我国的病例资源优势，建立多中心病例队列，开展多中心、前瞻性临床试验，在国际血液学界发出中国声音，提出CHIP和CCUS的中国解决方案，贡献中国智慧。

其四，国际大的血液肿瘤中心已开设了克隆门诊，由于CH除髓系肿瘤转化分险外，尚易发动脉粥样硬化从而心血管疾病发病率增高，此外，如果在CHIP基础上患有肿瘤需要放、化疗，其治疗相关性髓系肿瘤的发生率显著增高，因此，克隆门诊现阶段主要还是针对这些人群，常采用血液学、肿瘤学、心脑血管病学和遗传学专家共同参与的多学科协作（MTD）模式，近年MTD在我国已日臻完善，大型研究型医院应积极创造条件尽快开设克隆门诊。

其五，WHO（2022）已给出CHIP和CCUS的ICD编码，我国也应及时将这些编码加入我国的相应系统，这对于这些患者的诊治医保报销、规范管理、临床研究开展等都有非常重要的实用价值。

## References

[1] Xie M, Lu C, Wang J (2014). Age-related mutations associated with clonal hematopoietic expansion and malignancies[J]. Nat Med.

[2] Jaiswal S, Fontanillas P, Flannick J (2014). Age-related clonal hematopoiesis associated with adverse outcomes[J]. N Engl J Med.

[3] Genovese G, Kähler AK, Handsaker RE (2014). Clonal hematopoiesis and blood-cancer risk inferred from blood DNA sequence[J]. N Engl J Med.

[4] Khoury JD, Solary E, Abla O (2022). The 5th edition of the World Health Organization Classification of Haematolymphoid Tumours: Myeloid and Histiocytic/Dendritic Neoplasms[J]. Leukemia.

[5] Arber DA, Orazi A, Hasserjian RP (2022). International Consensus Classification of Myeloid Neoplasms and Acute Leukemias: integrating morphologic, clinical, and genomic data[J]. Blood.

[6] Valent P, Horny HP, Bennett JM (2007). Definitions and standards in the diagnosis and treatment of the myelodysplastic syndromes: Consensus statements and report from a working conference[J]. Leuk Res.

[7] Valent P, Orazi A, Steensma DP (2017). Proposed minimal diagnostic criteria for myelodysplastic syndromes (MDS) and potential pre-MDS conditions[J]. Oncotarget.

[8] Malcovati L, Gallì A, Travaglino E (2017). Clinical significance of somatic mutation in unexplained blood cytopenia[J]. Blood.

[9] Weeks LD, Niroula A, Neubrg D (2023). Prediction of risk for myeloid malignancy in clonal hematopoiesis[J]. NEJM Evid.

[10] Patel SA, Gerber WK, Zheng R (2024). Natural history of clonal haematopoiesis seen in real-world haematology settings[J]. Br J Haematol.

[11] Cargo C, Bernard E, Beinortas T (2024). Predicting cytopenias, progression, and survival in patients with clonal cytopenia of undetermined significance: a prospective cohort study[J]. Lancet Haematol.

[12] Gu M, Kovilakam SC, Dunn WG (2023). Multiparameter prediction of myeloid neoplasia risk[J]. Nat Genet.

[13] Xie Z, Komrokji R, Al Ali N (2024). Risk prediction for clonal cytopenia: multicenter real-world evidence[J]. Blood.

[14] Vlasschaert C, Mack T, Heimlich JB (2023). A practical approach to curate clonal hematopoiesis of indeterminate potential in human genetic data sets[J]. Blood.

[15] Woo J, Lu D, Lewandowski A (2023). Effects of IL-1β inhibition on anemia and clonal hematopoiesis in the randomized CANTOS trial[J]. Blood Adv.

[16] Xie Z, Fernandez J, Lasho T (2024). High-dose IV ascorbic acid therapy for patients with CCUS with TET2 mutations[J]. Blood.

[17] Haque T, Shastri A, Desai P (2024). A blueprint for pursuing therapeutic interventions and early phase clinical trials in clonal haematopoiesis[J]. Br J Haematol.

